# Corneal snowflake – a case of infectious crystalline keratopathy after penetrating keratoplasty

**DOI:** 10.1007/s15010-024-02383-x

**Published:** 2024-09-02

**Authors:** Colya N. Englisch, Tim Berger, Sören L. Becker, Berthold Seitz

**Affiliations:** 1 Department of Ophthalmology, Saarland University Medical Center, Kirrberger Strasse 100, 66421 Homburg/Saar, Germany; 2 Department of Experimental Ophthalmology, Saarland University, 66421 Homburg, Germany; 3 Institute of Medical Microbiology and Hygiene, Saarland University, 66421 Homburg, Germany

A 60-year-old male corneal transplant recipient presented with an asymptomatic central snowflake-shaped lesion of the graft on a control visit three months after a penetrating keratoplasty (PKP) of the left eye due to keratoconus. On a previous control visit, six weeks after PKP, anterior segment examination had been uneventful without any epithelial defects except signs of moderate dryness. Decreased vision, conjunctival injection, or foreign body sensation were not reported. There was no medical history of other corneal conditions including infectious keratitis.

Slit lamp examination demonstrated a grey-white branching opacity in the superficial corneal stroma without epithelial defects or signs of surrounding or anterior chamber inflammation (Fig. 1A and B). Anterior segment optical coherence tomography did not allow for clear discrimination between the infectious lesion and normal corneal stroma. Microbiological analysis (agar plate culture and broad-range 16 S polymerase chain reaction) of the corneal scraping samples did not reveal any pathogens.

An empirical topical anti-infectious treatment was initiated, consisting of 0.5% moxifloxacin and 5% vancomycin eye drops alternating hourly for one week, followed by 0.5% moxifloxacin eye drops five times daily for four weeks. Topical immunosuppression scheme (0.5% loteprednol etabonate eye drops five times daily reduced by one drop every 8 weeks after keratoplasty for a transplant diameter of 8.5 mm) was not changed to prevent corneal graft rejection. After one-month of anti-infectious treatment, the clinical finding was stable without improvement (Fig. 1C).

Infectious crystalline keratopathy (ICK) is a rare typically asymptomatic complication after keratoplasty and is mainly observed in patients on topical steroid therapy. Under these conditions, pathogen entrance and proliferation in the corneal stroma are facilitated. Epithelial defects (e.g., due to loosening of a keratoplasty suture) are often critical in this context [1]. ICK is a true challenge from a diagnostical and therapeutical perspective [1, 2]. Isolation of microorganisms in ICK is rarely successful from corneal scraping samples but instead mostly obtained from invasive corneal biopsies [1].

There is a number of species causative of ICK including bacteria with viridans streptococci being most frequently encountered [1]. In rare cases, ICK has also been described in fungal keratitis [1, 3]. Nevertheless, ICK remains often refractory to topical anti-infectious treatment, which has been associated with biofilm formation, which is a characteristic microscopic finding [1]. Corneal surgery (e.g., lamellar keratectomy) is often required to treat refractory courses, with PKP remaining the ultima ratio [1].

ICK is a rare but important differential diagnosis to microbial keratitis, corneal dystrophies, and more [1]. In contrast to typical microbial keratitis with anterior segment inflammation, ICK is regularly silent and thus diagnosed lately [1]. Hence, this case of impressive snowflake-shaped ICK ultimately highlights the importance of regularly examining patients after PKP, especially when topical steroids are used and keratoplasty sutures are still in place.

**Figure 1:**
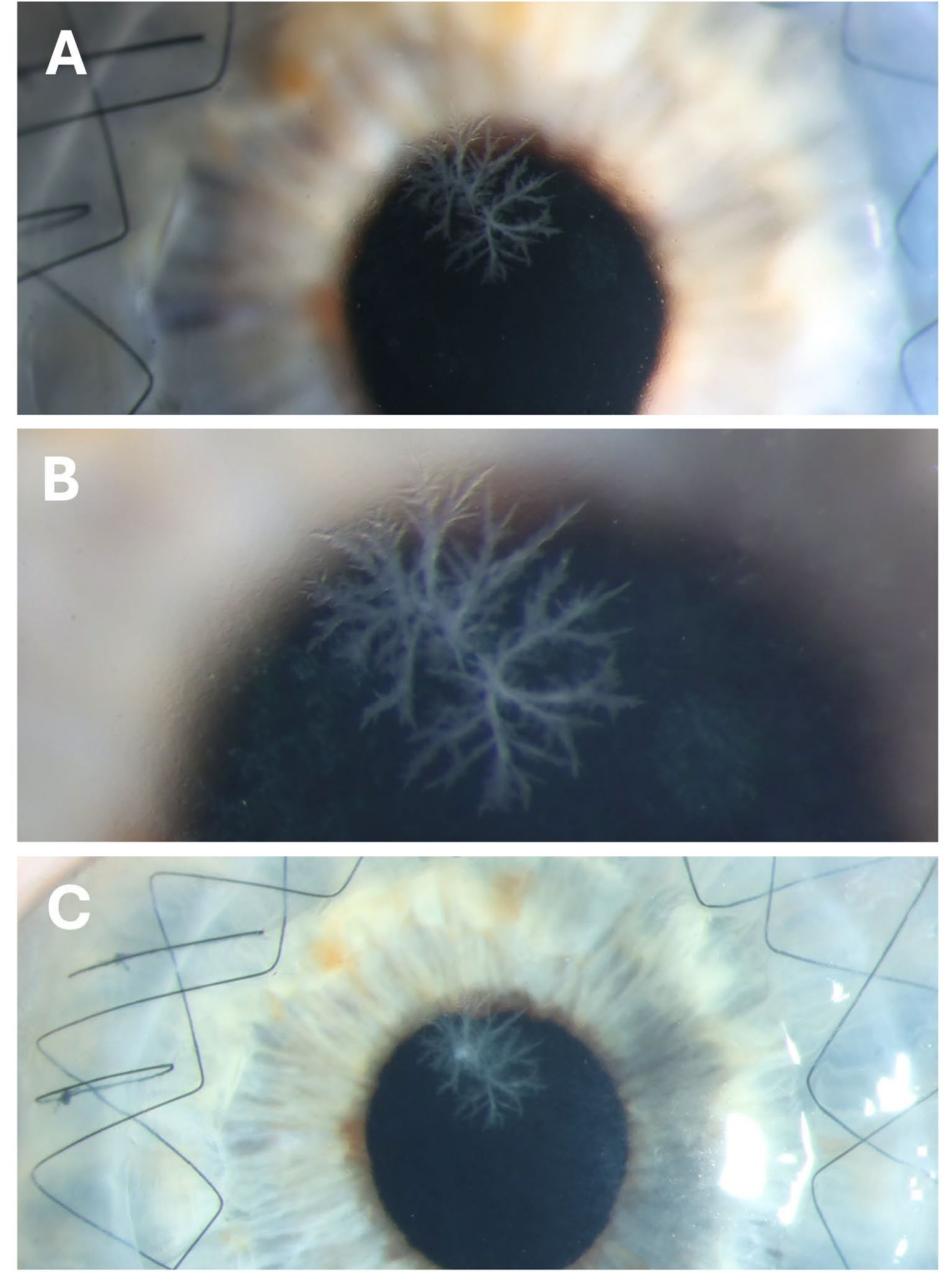
Figure 1

## Data Availability

No datasets were generated or analysed during the current study.
